# A qualitative study of cultural concepts of distress among Rohingya refugees in Cox’s Bazar, Bangladesh

**DOI:** 10.1186/s13031-024-00606-4

**Published:** 2024-07-30

**Authors:** Kathy Trang, Caroline Hiott, A. K. Rahim, Shafiqur Rahman, Alice J. Wuermli

**Affiliations:** 1 Department of Epidemiology, Harvard TH Chan School of Public Health, 677 Huntington Ave, Boston, MA 02215 USA; 2 Global TIES for Children, New York University, New York, NY USA; 3 Humanitarian Assistance Program, Cox’s Bazar, Bangladesh

## Abstract

**Background:**

Rohingya refugees residing in Bangladesh have been exposed to profound trauma in addition to ongoing daily stressors of living in the refugee camps. Accurate assessments of mental health burden and their impact among this population require culturally sensitive tools that remain lacking in this context. The purpose of this study was to characterize salient cultural concepts of distress (CCDs), their causes, consequences, and approaches to treatment, among Rohingya refugees living in Cox’s Bazar, Bangladesh, to help inform future measurement and intervention design.

**Methods:**

Between December 2020 and March 2022, 106 free-listing interviews and 10 key informant interviews were conducted with community members to identify and better understand common CCDs. Rohingya research staff analyzed the interview transcripts by tabulating the frequency of unique CCDs in the free-listing interviews and the unique attributed causes, signs, consequences, and treatment strategies for each CCD in the key informant interviews.

**Results:**

In total, five CCDs were identified: *tenshon* (tension), *bishi sinta* (excessive thinking), *feshar* (pressure), *gum zai nofara* (unable to sleep), and *shoit-shoit lagon* (feeling restless and/or trapped). Although the five CCDs had overlapping symptoms, they also had unique presentation, consequences, and preferred strategies for treatment that may impact service-seeking behavior. Three out of the five CCDs were considered life-threatening, if severe and left untreated.

**Conclusion:**

The five CCDs identified are culturally salient ways of experiencing and communicating distress within this community but are not adequately captured in existing mental health assessments for this population. This may negatively impact programmatic efforts among the group.

## Introduction

One of five individuals affected by armed conflict and war are estimated to have a common mental disorder [8]. These estimates are similar, if not higher, to those observed among Rohingya refugees in Cox’s Bazar, Bangladesh [22, 35, 37, 42]. Driven from Myanmar in the context of genocide in multiple waves over several decades—the most recent and largest of which in August of 2017—approximately 954,000 Rohingya currently reside in crowded, makeshift camps on the southeastern coast of Bangladesh [48]. The group constitutes the highest number worldwide of what the United Nations High Commissioner for Refugees has termed “stateless people” [25]. Individuals are distributed across 34 camps in Cox’s Bazar, the largest of which, Kutupalong, has an average population density of 50,299 persons per km^2^, and is the most populous refugee camp in the world [49].

Trauma exposure among this population is high with 99% of individuals reporting exposure to gunfire, 93% reporting destruction of homes, 56% torture, and 33% sexual assault [41]. Nearly one third of adults also report experiencing physical abuse from a spouse or family member [42]. These traumatic experiences are compounded by the daily stressors of living in the camps, ranging from difficulties with sufficient income (95%), food (79%), and access to education (72%), to problems with living space (62%), sanitation facilities (62%), physical health (62%), and water (60%) [41]. During the COVID-19 pandemic, heavy restrictions were placed on normal activities and programs in the camps (i.e., education, livelihood), while flooding and fires continue to destroy homes and displace families. Additionally, over 25,000 Rohingya have been relocated to Bhasan Char, a controversial, newly developed island prone to flooding in the Bay of Bengal [26]. All these daily stressors are intensified by fears for safety and security, particularly for young women, as armed groups and incidences of violence and trafficking rise [1]. It is no surprise then that a cross-sectional survey of two Rohingya camps found that only 5% of participants rated their subjective quality of life as either good or better [23].

Mental health infrastructure remains limited in Bangladesh generally and in the camps specifically. To meet mental health needs within the camps, mental health and psychosocial support (MHPSS) services have been integrated into basic health services in accordance with the WHO Mental Health Gap Action Program (mhGAP) guidelines [2]. As of October 2019, approximately 81% of primary care centers within the camps had at least one staff trained on mhGAP and in 2022 there were 10 MHPSS Centers in the camps [15]. At the same time, overall uptake of MHPSS services remains low [47]. This may be driven by disparities between how mental illness is assessed and treated in these programs and local understandings of mental distress, including how it is communicated about and treated within the community [9, 47].

In the context of Rohingya refugees living in Cox’s Bazar, there have been at least a dozen studies looking at the mental health burden in this population [9, 11, 13, 22, 27, 30, 34, 35, 38, 41–43, 47]. These studies have used a range of standardized, internationally used screening and diagnostic instruments, including: the Harvard Trauma Questionnaire (HTQ, [37, 42], Hopkins Symptom Checklist (HSCL; [41], Patient Health Questionnaire-9 [30, 37], Generalized Anxiety Disorder-7 (GAD-7); Refugee Mental Health Assessment Package [47], Refugee Health Screener [38], Impact of Event Scale–Revised [22], Geriatric Depression Scale [35], Centre for Epidemiologic Studies Depression scale [11], the International Depression Symptom Scale [36], and Kessler-6 [36]. Most studies acknowledge the importance of cultural adaptation and a thorough validation of the instruments before use. Unfortunately, resources (time, financial, human) often limit the extent to which researchers can engage in this process, and adaptation varies drastically from study to study. Commonly, studies have relied on forward and backward translation to adapt standardized measures to the local context [37]. Many mental health assessments used are developed and normed outside of majority world countries and populations [16]. But the degree to which these measures can validly assess mental distress in culturally diverse populations is unclear. The symptoms these measures assess may not represent the most salient ways in which distress manifests, or how people express their distress in a particular context, resulting in the problem of category truncation [21]. Inappropriate use of measures can skew estimates of mental health epidemiology with negative downstream effects on efforts to detect and treat cases for common mental disorders [47].

Accurate assessment of mental health burden and their impact in the Rohingya refugee context necessitates understanding local cultural concepts of distress (CCDs), which refer to a spectrum of distress ranging from everyday concerns to severe psychopathology [32]. CCDs encompass both idioms of distress, or local ways of expressing everyday concerns, and cultural syndromes, or co-occurring symptoms with a distinctive temporal course, severity, and responsiveness to treatment. Among Rohingya communities, characterization of CCDs can facilitate the cultural adaptation of screening measures and interventions to be better aligned with local conceptualizations of distress, their causes, and appropriate treatment. As Tay and colleagues (2019) have reported, many Rohingya refugees in Bangladesh are reluctant to seek out mental health services because they are both unfamiliar with what services are available and because the CCDs and the explanatory models that make sense to their lived experiences —linking brain (*mogos/demak*), mind (*dil/mon*), soul (*jaan/foran*), and the physical body (*jism/gaa/shoril*)—are often not recognized by outside providers or adequately captured by translated measures [13].

Empirical investigation into Rohingya CCDs remains limited. Through a review of the literature, Sudheer and Banerjee [45] have pinpointed a number of general health, body-, brain-, cognitive- (e.g., for attention), and emotion-related (e.g., for anger) terms Rohingya use to communicate about the physical and mental distress they may experience. Characterization of how those terms are used in everyday life to talk about distress remains limited. As Sudheer and Banejee [45] pointed out, much of the extant literature has focused predominantly on trauma and PTSD with comparatively little attention paid to subsyndromal mental health and somatic complaints emerging from daily stressors,even fewer of such investigations have been qualitative. As Tay and colleagues (2019) have argued, there is a present need to understand Rohingya CCDs and the explanatory models surrounding them (what causes the symptoms or illness, what those symptoms or illnesses impact, how and why they should be treated) because these manifestations of distress cannot be assumed to be equivalent to psychological concepts of depression, anxiety, or PTSD. Thus far, some initial work to develop local assessments of CCDs has been undertaken by Riley and colleagues [42] to create a Somatic Symptom Scale and a Local Idioms of Distress Scale based on interviews with Ministry of Health staff and mental health professionals working in the region. To the best of our knowledge, however, no study has attempted to comprehensively and systematically map and structure CCDs that exist within this community, and explore in depth their cause, consequences, and approaches to treatment.

Our qualitative study thus aimed to identify common CCDs and characterize their causes, phenomenology, impacts on functioning, and treatment among a community sample of Rohingya refugees in Cox’s Bazar, Bangladesh. The findings of this qualitative study can guide future adaptation or development of standardized mental health measures and intervention design and implementation among the Rohingya communities in Cox’s Bazar.

## Materials and methods

### Setting and sample

The core research team consisted of three US-based researchers and a field team of ten (*n* = 9 enumerators, *n* = 1 team lead), multilingual Rohingya research staff recruited from a community-based social enterprise established in response to the 2017 influx of Rohingya refugees in Cox’s Bazar. The community-based organization often partners with organizations and institutions who seek on-the-ground partnerships, project consultations, or assistance with data collection. The field staff from the organization lived and worked within the refugee camps.

Free-listing and key informant interviews were conducted in 2 of the 34 refugee camps in Cox’s Bazar between December 2020 and March 2022. These two camps were chosen because of the field team’s established presence there, which allowed the project to leverage and expand on the rapport that existed between the community and the community-based organization. It also helped reduce the opportunity for rumors to spread—a not uncommon hurdle in conflict-affected contexts. This was additionally advantageous for recruiting participants as mobility was limited due to the COVID-19 pandemic.

For all activities, individuals were eligible for participation if they were Rohingya, at least 15 years old, and living in the refugee camps. Study recruitment was divided into two phases: free-listing and key informant interviews. For the first phase of the study (free listing), participants were recruited by convenience sampling across two camps. Research staff recruited participants in public locations within the camps. Additionally, research staff also approached *mahjjis*, who are a type of community leaders, for recommendations on whom to approach. For the second phase of the study (key informant interviews), participants were recruited based on recommendations provided during free listing interviews.

All interviews were conducted face-to-face in Rohingya at the participant’s home or in a nearby location. Interviews were audio-recorded with consent then transcribed and phoneticized in Latin script by the field team. Analyses were conducted in the local language, primarily to tabulate the frequency of unique CCDs and their perceived causes, signs and symptoms, effects, and treatment. Sections "Free Listing Interviews" and "Key Informant Interviews" provide more details on the data collection and analysis steps for the free-listing and key informant interviews, respectively. Throughout the data collection and analysis process, the field team met frequently with the US team to troubleshoot and discuss and critically inspect emerging findings. Due to the COVID-19 pandemic, all research training and coordination occurred virtually.

Research ethics review was obtained by the Institutional Review Boards (IRB) for New York University (IRB-FY2021-4875). This IRB issued a reliance agreement for the Humanitarian Assistance Program, which is a Bangladesh-based organization that did not have an internal IRB but who had local experts to review and approve the study. All research staff were provided with training on ethical conduct, qualitative research methods, and strategies to mitigate psychological distress and to ensure confidentiality. Research staff read the consent forms to prospective participants, as many were illiterate [24], and provided opportunities for participants to ask questions. Participants were asked to provide verbal consent.

### Free listing interviews

CCDs were first identified through free listing interviews, which is a method of structured interviewing that asks broad questions with the intent of generating a list of items that reflects community-shared experiences. Example questions included “When everything in your household/family/life is going well, what kind of mental and physical feelings do you have?” and “When everything in your household/family/life is going poorly, what kind of mental and physical feelings do you have?” For each of these questions, participants were asked to reflect not only on their own experiences but also from observations they had of others around them. Questions were co-generated and co-translated with multilingual Rohingya research staff and a US-based researcher with working knowledge of the Rohingya language. Because participants were asked to reflect on community perceptions rather than their own, the method helps to minimize issues associated with convenience sampling [5].

Interviewees were a convenience sample, representing the target population for future research and programming. They were those who met study criteria, were available during recruitment, and were willing to participate. Specifically, to recruit participants, the field team conducted walks around the two camps, starting at multiple locations centrally located within the camps and taking different routes each day; they attempted to recruit those they encountered. The recruitment period spanned 11 days. The field team enlisted the help of mahjis to recruit women who were home and likely to have time to participate in the interviews. A target sample size of 80 was chosen as a sample size of 40 per sub-group is typically needed to establish whether there is cultural agreement/consensus on a given topic domain (e.g., what constitutes mental wellbeing); because we expected some potential differences between men and women, a minimum sample size of 80 was necessary. In total, 106 free listing participants were recruited. This sample comprised 62 men and 44 women, ranging ages 19 to 39. Men were oversampled because more men were outside of their homes than were women.

Interviews were then transcribed and analyzed by the local research team to generate a table of CCDs mentioned by participants, as well as the frequency with which they were mentioned. This process involved the local team working in pairs to identify unique items from the free-listing interviews and combining those that were conceptually similar (e.g., having head pain and headache). Any disagreements between pairs were discussed and settled by a third party. At the end of the analysis, a list of priority problems—that is, those mentioned by at least two participants and appeared to be severe in terms of impact but not yet well understood by the study team—were identified and retained for further exploration in key informant interviews.

### Key informant interviews

The field team then contacted and interviewed 10 key informants identified as cultural experts in the free-listing exercise. These individuals were often community leaders and older community or family members. Interviews with these individuals explored the symptoms associated with each of the major CCDs identified in the free listing, their perceived causes, effects on the person with the problem and those around them, and what people currently do versus what they would do if they had resources available to treat the problem. Again, the local research team transcribed the interviews and synthesized the transcripts using the same frequency-based approach as the free listing, generating a summary sheet for each CCD. They then identified representative quotes characterizing key informants’ beliefs about their causes, lived experience, impacts, and treatment.

## Results

Free listing activities generated a list of 25 terms to describe mental health and distress symptoms and attributes. Follow-up interviews with key informants to better understand the underlying conditions identified five primary cultural concepts of distress: *tenshon* (tension), *bishi sinta* (excessive thinking/worrying), *feshar* (pressure), *gum zai nofara* (unable to sleep/insomnia), and *shoit-shoit lagon* (feeling restless and/or trapped).

### Tenshon

*Tenshon*—derived from and loosely defined by the English word “tension”—is a widely understood idiom of distress that the majority of our participants reported either experiencing personally or knowing someone close to them who has experienced it. Like its English etymological origin, the word *tenshon* is often used to denote psychological tension, or what is commonly referred to as “stress” in everyday English.

Someone with *tenshon* will often have a headache (*matha horani*) or sharp pain (*gaa sissiya*) in their upper body. One key informant (male, 34) suggested that *tenshon* had a proliferating nature; that is, the psychosomatic symptoms may spread from the initial point of entry to other parts of the body: “All you need is a little bit of *tenshon* to enter the body… then it’ll keep increasing.” Due to the headache and/or bodily pain associated with *tenshon*, those afflicted will often feel weak and tired. They might also appear as though they are sulking (*mukh hala*), want to “go somewhere far and sit there” (female, 25), or have a bad temperament (*guishsha ba mezas horaf*). Other common symptoms include excessive anger (*manuish loi-guishsha*), pressure (*feshar*), excessive thinking/worrying (*bishi sinta*), restlessness (*foran shoit-shoit gora*), insomnia (*gum zai no-fara*), loss of appetite (*haana-fina hai no fara*), and self-isolation or reticence (*gorot boi taka ba manuish loi hota no huwa*).

Causes of *tenshon* are wide-ranging and include forced removal from homeland, family and traditional living arrangements, conflicts within the household or with neighbors, and financial and employment concerns. Only women in our sample reported the inability to fulfill culturally expected roles for women as a cause of *tenshon*. One participant (female, 25), for instance, associated *tenshon* with becoming a new mother.

The gravity and perceived harmfulness of *tenshon* is widely understood in the Rohingya community. Generally, a person will not be able to work or look after their children well. In some cases, *tenshon* can also be more serious. One key informant (female, 50) expressed this by relating the impact of *tenshon* to her understanding of Rohingya folk-anatomy. In the Rohingya language, the word *hoilla* is sometimes used for both the liver and the heart and is also used to denote the source of certain emotions. She said, when you have *tenshon*, “Your *hoilla* gets dirty (…). You can die from it.” Another participant also mentioned death as a result of untreated *tenshon*. Even though *tenshon* was often described using psychological symptoms, participants stressed that prolonged periods of *tenshon* can lead to physical illnesses, damaging internal organs and subsequently leading to death.

Participants listed several strategies for managing *tenshon*, including: meeting with or chit-chatting with others (*manush loi moshuwara gora ba manuish loi gof-sof gora*), seeking medical help (*dattoror hase za*), and taking medicine (*dabai haa*). Although interpersonal strategies for managing *tenshon* were commonly named, others thought the condition could also be self-managed through rest, changes in diet, or travel to somewhere “you can feel the breeze” (male, “elder”).

### Bishi sinta

*Bishi Sinta* is best translated in English as “thinking a lot” or excessive worrying. The word *sinta* on its own in Rohingya (and other Indic languages that derive the term from Sanskrit *chinta*) is used to denote both general thought (mentation) and worries. To differentiate between neutral, general thoughts and negative thoughts (worries), participants will often say *sinta-bafona* for the former and *horaf sinta* for the latter. However, when used with the word *bishi*, meaning “excessive”, the phrase *bishi sinta* aligns more closely with the English concept of “excessive worrying.”

Common symptoms of *bishi sinta* identified by participants included loss of appetite (*haana-fina hai nofara*), insomnia (*gum zai no fara*), not wanting to work (*ham-horos gorito mone no huwa*), high blood pressure (*feshar baron*), feeling physically weak or unwell (*gaar oshanthi*), and having sudden sharp pain or flashbacks (*demak sho'ot oi zon goi*). *Bishi sinta* can lead to rumination a person becoming unusually quiet and withdrawn.

Common causes of *bishi sinta* often overlapped with or exacerbated their perceived impacts. Specifically, participants thought that *bishi sinta* could be caused by having frequent quarrels with family members (*goror manuish loi hoijja*) or feeling dissatisfied and distant from their children (*fuwain-saindore gom no laga*). Other important causes included having a lack of work and/or financial resources. For some people, ruminating on the state of their refugee status (e.g., wanting to go back to Myanmar; being stateless) can also trigger *bishi sinta*. Notably, *tenshon* can cause *bishi sinta;* inversely*, bishi sinta* can cause both *tenshon* and *feshar*. In fact, *tenshon* and *bishi sinta* are so closely aligned that they were often used interchangeably by participants to express different degrees of worry. Thus, participants often referred to the cyclic nature of the condition.

Impacts of experiencing *bishi sinta* include feeling physically weak (*gaa komzur uwa*), experiencing weight loss (*ledha oi zon goi*), and not being able to speak to others (*manuish loi hotha no huwa*). One participant (male, 52) explained the social repercussions of this CCD, saying, “*Bishi sinta* can lead to laziness. People won’t think too highly of a person that becomes lazy.” If severe or prolonged, *bishi sinta* is also commonly thought to cause brain/mind damage (*damak sho'ot oi zon goi*) and heart damage (*dil’oth shomoshsha*).

To treat *bishi sinta*, participants thought that people could socialize or gossip with others (*maniush loi moshuwara gori le*), try to sleep more (*gum za foribo*), seek the help of a doctor or hospital (*dattoror hase ba datta-hanat zon*), take medicine (*dabai-oshud haa foribo*), or seek work (*ham gori bolla rasta tuwa*) and earn more money (*tia-foisha hamon*). One key informant (male, 30) reported that a person should go to a *boddo*, or traditional healer, for treatment, but did not elaborate on any treatments given by the *boddo*.

### Feshar

*Feshar*—derived from and loosely defined as the English word pressure—is often associated with neck pain that climbs upward from the neck, causing an increasingly tense sensation in the sufferer’s face. The person becomes very weak and lethargic as a result. They are also said to be extremely irritable and prone to acting out. Common symptoms include neck pain (*goddona sissiya*), sharp body pain (*gaa sissiya*), headache (*matha horani*), face swelling (*muk fuli zon goi*), being upset or unhappy (*oshanti laga*), and feeling numb (*besuth oi zon goi*).

*Feshar* can either result from or cause *tenshon* and *bishi sinta*. Financial concerns (*tiya foisha loi sinta*), insomnia (*gum zai no fara*), conflicts within the household (*goror mainshor loi hoijja*), or the perceived and/or actual existence of blood problems (*lou horaf oile / lou nosoli le*) can also trigger *feshar*.

Most participants thought that *feshar* will affect an individual’s ability to take care of their children (*fuain sñoli no fara*), manage household tasks such as cooking (*goror ham gori no fara*), and socialize with others (*mainshor lo hotha-bathra hon*). If severe, *feshar* can cause a shock to the mind/brain (*demak sho’ot oith fare*) and even death (*mori zaigoith fare*). A key informant (male, “elder”) highlights this by saying, “In my understanding, there are a few types of *feshar*. I can clearly think of two types… One is when your face is swollen. And the other is when your neck aches. They say not to press your neck. Or else the nerve will rip, and you will die.” Other key informants also voiced that if *feshar* reached the head and was severe enough, a person could die.

To treat *feshar,* there was consensus that people will “try to bathe first… then apply fresh garlic paste. Or paste made from tree barks and leaves. Things like that help a little bit […] you still need medicine” (female, 25). In addition to paste and balms made from garlic, bark, and other leaves, people will consume *ro'un haa* (garlic) and *dñuir-gula* (a wild, tropical fig). A key informant shared that it was important to try to sleep more (*gum za foribo*), as the pressure and “sadness will go away” when sleeping (male, 37). Like *tenshon* and *bishi sinta,* people with *Feshar* may also seek help from a doctor or take medicine. However, unlike the first two conditions, people are much less likely to think that socializing or discussing the problem with others will help alleviate their symptoms.

### Gum zai no fara

*Gum zai no fara,* meaning not being able to sleep*,* is associated with feeling upset, anxious, and fatigued. The severity of the condition is communicated by participants using such expressions as “nose and face turning red” (*nak muk lal oi za goi*), which convey how upset the person is by their condition. The person’s fatigue may lead to dizziness (*gaa zimon*) and headaches (*matha hñoron*).

A person who suffers *gum jai no fara* is thought to struggle with taking care of their children (*fuain-suaindor kermos gori nofara*) and with doing everyday work (*ham-horos gori no fara*). The condition is also thought to affect the body (*gaalla nuksan uwa*), causing those afflicted to feel unwell physically (*gaath oshanthi laga*) and experience either headaches (*matha tontona*) or other unwanted head-related sensations (*matha tik no thaka*). Participants also frequently drew connections between this condition and other CCDs identified. For example, one key informant (male, 30) shared, “If you cannot sleep at night, you will get *tenshon*. You will get *bishi sinta*. You will get diseases of the body. They are all related.” Another key informant (male, 34) also mentioned that *gum zai no fara* is connected to other CCDs, saying, “We can often tell when someone else can’t sleep. They may have *tenshon*, they may have *feshar…* we can tell.”

The main causes of this CCD are tension (*tenshon*), excessive thinking/worrying (*bishi sinta*), pressure (*feshar*) and other illnesses *(oshuk uwa)*, as well as a lack of food before sleep (*bath hai no farile o gum zai no fara*). Common treatments mentioned for *gum zai no fara* included going to the hospital or doctor (*datta-hanat/dattoror hase za*) and taking medicine (*dabai/oshud haa*). Some participants also mentioned treating the condition by discussing their problems with other people (*manuish loi moshuwara gora*), sleeping under a fan (*fenor bathaish hai gum za*), or simply sleeping more (*gum zai fara*).

### Shoit-shoit lagon

*Shoit-shoit lagon*, meaning to feel restless and/or trapped, is often described as feeling upset and anxious, hopeless about one’s current living situation (that is, trapped within the camps), and sometimes excluded when seeing others moving freely outside.

*Shoit-shoit lagon* can have both physical and psychological causes. It is often experienced because of external, environmental factors such as the congested conditions of the shelters and the overwhelming heat in the camps. It can be felt psychologically due to the strain of familial contentions (*gorot obafot thaile)*, quarrels within the home *(bow fuian loi hoijja dile*), or not having enough money (*tiya foisha no thakile*).

A person suffering from *shoit-shoit lagon* may struggle to sleep (*gum zai no fara*), feel unwell in the body (*gaat maze oshanti laga*), and experience difficulty working, eating, or caring for their children (*hono haam-horos gori no fara, hana hai no fara, fuain suain sai no fara*). Experiencing *shoit-shoit lagon* can also trigger an overwhelming desire to escape or flee, particularly among women, who are often restricted to indoor spaces due to the cultural practice of *purdah*, or segregation of genders. Rohingya in this context have a synonymous term for this condition: *goroth thakito mone no huwa*, meaning “not wanting to stay at home”.

Although men can, and often do, leave their shelters, they too can feel *shoit-shoit*. A female key informant (25) described this condition inflicting her husband, explaining, “He may come home. But then after a few minutes, he leaves again to go to a store. He’ll come back again, but then quickly leave to chat with other people. I can tell he’s feeling *shoit-shoit*.”

Participants did not associate prolonged, untreated *shoit-shoit* with the same adverse consequences associated with the other CCDs but did believe that it can contribute towards the development of those conditions over time. The most reported treatment for *shoit-shoit* was to go the doctor (*dattaror hase zaa*). People also mentioned taking medicines (*dabai-oshud haa*), discussing their problems or taking advice from others (*manuish ottu nosiyot luwa, moshuwra gora)*, sleeping more (*gum zai fara*), and trying to better get along with their children (*fuaindor loi kushi taka*) to reduce *shoit-shoit*.

## Discussion

This study begins to fill gaps previously identified by Sudheer and Banerjee [46] and Elshazly et al. [13] on how to assess the burden of mental health appropriately and sensitively among Rohingya communities residing in the refugee camps of Cox’s Bazar, Bangladesh. Through qualitative interviews conducted with members of the community, including individuals who were considered cultural experts, this study identified five cultural concepts of distress (CCDs): *tenshon* (tension), *bishi sinta* (excessive thinking/worrying), *feshar* (pressure), *gum jai no fare* (unable to sleep/insomnia), and *shoit-shoit lagon* (feeling restless/trapped). These CCDs were widely understood and used by participants to communicate the distress they experienced because of past and ongoing stressors in their lives. Although all CCDs were thought to originate out of mundane conditions—rather than major traumatic events—three of the CCDs (*tenshon, bishi sinta,* and *feshar*), when severe and left untreated, were thought to be fatal. Although some conditions (e.g., *tenshon*) were considered treatable by confiding in others, others (e.g., *feshar*) were not thought of as responsive to social intervention.

*Tenshon* and *bishi sinta* are two well-documented CCDs among South and Southeast Asian populations [28, 52]. Specifically, a recent systematic review of studies documenting *tenshon* among South Asian populations yielded 122 reports of studies primarily in India, but also included studies in Nepal, Bangladesh, and among the South Asian diaspora in several other countries [52]. Across those reports, the most frequently mentioned causes of *tenshon* were resource scarcity, inability to fulfill socially and culturally expected roles (e.g., being able to provide for the family, marry one’s child), and gender role-related stress. Although both men and women were thought to be vulnerable to *tenshon,* the causes of and ways in which people reported coping with tension were thought to differ. In our study, only female key informants definitively verbalized that being unable to fulfill culturally expected roles was a cause of *tenshon*, however, both female and male participants reported that loss of traditional living arrangements caused *tenshon*. Furthermore, some gender differences were observed in the terms people used to communicate similar experiences of distress. Compared to women, Rohingya men, in our experience, were more likely to have adopted vocabulary from other languages into their vernacular as they more commonly interact with Bangla, Chittagonian, and English speakers. Women in this context have fewer interactions outside of their households. This suggests that although vulnerability to tension may be similar among men and women, the way it manifests, is communicated about, and is coped with may differ. The linguistic issues observed in the context of this study are likely to generalize and should be considered when designing or disseminating mental health and psychosocial support materials to Rohingya men and women.

Similar to our study, the systematic review conducted by Weaver & Karasz [52] found that most studies identified both a mild form of tension that alleviated itself in a span of days or weeks and a prolonged form of tension that was perceived to be associated with increased morbidity and mortality. Most participants in our study, similar to what has found in the wider literature (e.g., [6, 7, 14], believed tension could be coped with and treated through individual and interpersonal coping strategies, such as changing their diet or seeking social support from others. Notably, caring for and getting along with one's children (or inversely, not being able to care for or get along with one’s children) is an important cause (*tenshon*, *bishi sinta*), result of (*feshar, gum jai no fare, shoit-shoit lagon*) and treatment (*shoit-shoit lagon*) for all the identified CCDs. Where our findings diverge is that our participants also commonly endorse turning to doctors for treatment. However, it is unclear from the interviews who is included within the category of “doctor” and relatedly, what under the broad category of “medicine.” Doctors in this context could reference multiple types of traditional and informal healers, including spiritual healers (bouid-dou), religious scholars (fou-yirr), Quran reciters (mou-loi/habés), and unlicensed individuals prescribing Western medicine (daac-torr) [47]. A health literacy survey of 1634 Rohingya refugees in Cox’s Bazar found, for instance, that over half of participants reported first seeking the assistance of a village doctor (that is, traditional healer) when a family member is ill before turning to a pharmacy or government hospital [40]. Most further report feeling dissatisfied with treatment at formal medical establishments and poor uptake of mental health and psychosocial support services [13]. Future studies should clarify the folk and biomedical services Rohingya persons preferentially use to treat conditions like *tenshon* and what medication they use at varying levels of severity and differences based on sex and age.

*Bishi sinta*, or “thinking a lot,” is also well documented in the global mental health literature with one systematic review finding over 138 relevant studies [28]. This included 41 studies reporting on populations in Southeast Asia and 12 studies, on populations in South Asia. Thinking a lot is also one of nine CCDs recognized in the Diagnostic Statistical Manual-5 [4]. This CCD generally references intrusive, unwanted thoughts but the content and manifestation of thinking a lot has also been shown to demonstrate considerable cross-cultural difference and to capture experiences of distress beyond depression, anxiety, or PTSD, although symptoms commonly overlap [28]. Most commonly, thinking a lot has been found in the wider literature to emerge from strains in social relationships, economic or structural hardships, and exposure to adversity. Some have argued that the distress and social stigma associated with “thinking a lot” emerges from its deviation from what can be considered culturally an ideal state of mind. Specifically, some studies focused on Southeast Asian populations have situated “thinking a lot” within a broader cultural context that values mindfulness [19], stoicism amidst adversity [33], and detachment from worldly concerns [12]. In such contexts, thinking a lot may be perceived as spiritual weakness and an inability to maintain inner harmony. Future work should examine how *bishi sinta* is situated within broader cultural and religious belief systems Rohingya persons may have regarding how to regulate and maintain ideal mental or emotional states.

Although less has been written about *feshar*, or pressure, somatic complaints are not uncommon [17, 44]. Of the five identified CCDs, *feshar* may be the condition most defined by somatic complaints, although such complaints are present in all five CCDs. From the descriptions participants provided, *feshar* does not appear to be dissimilar to “khyâl attacks” experienced by rural Cambodians, which are described as when blood and khyâl (wind-like substance) surge upwards through the body [17], neck stiffness and fear of rupture of blood vessels in the neck are thought to be caused by khyâl. For rural Cambodians, neck soreness and pain are thought to be somatic expressions of physical trauma and poor health [20]. In our study, *feshar* was similarly thought to be indicated by neck pain, bodily pain, and headache. Future studies should examine how these somatic complaints associated with *feshar* may map onto physical and mental health issues.

Across the interviews, what is notably absent are references to religious or supernatural causes. Past studies have indicated that Rohingya persons often fit interpretations of their physical and mental health within a framework of Islamic beliefs and traditions [47]. Outside of this study, our preliminary work with this population indicates that Islamic beliefs and traditions—including *taqdir* (God’s will), *gunah ar sawab* (sins and good deeds), and *purdah* (gender segregation)—are pervasive concepts that Rohingya adhere to and enact in their daily lives. It is possible that participants in this study may not have mentioned supernatural or religious causes as readily because of the sensitivity of talking about these topics in the context of mental health, particularly in front of people other than their close family members and friends, the phrasing of the questions in the study; or the stigma associated with seeking treatment from traditional healers, as more religious segments of Rohingya society may regard such treatments as bordering sorcery. It may also be that these conditions may not carry the same religious or supernatural significance as others, or that they are not commonly caused by religious or supernatural factors. Riley and colleagues [42] had assessed three local idioms of distress based on Prosser’s (2006) work, focusing on whether participants believed they were under a spell, possessed by a bad spirit/demon, or under the control of black magic. They had found relatively low levels of endorsement with 10% or fewer participants endorsing these experiences over the past month.

Future studies should examine how the perceived etiology, phenomenology, and treatment of CCDs relate to wider religious and supernatural belief systems within the Rohingya community. Although religion can strengthen coping resources [39], it can also be a source of stress and a barrier to treatment. Among Muslim communities in particular, notions of *sin* and shame, which often stem from religious beliefs, can be major sources of psychological distress [3]. Furthermore, other studies have found that Rohingya persons may sometimes attribute mental illness to malevolent and benevolent spirits (*jinn-fori*), evil eye (*nozor),* and spiritual possessions (*asore faiyi*) [47], although it is unclear whether the CCDs identified within the context of this study carry the same spiritual significance. Future studies should thus explore how religion and spirituality serve as both resources and stressors within this community and how and to what degree they can be integrated into MHPSS programming to strengthen uptake and effectiveness.

Increasingly, scholars have argued for the integration of CCDs into mental health assessments and interventions [10]. This is motivated by the limited ability of standardized mental health assessments to adequately capture the ways in which distress is experienced and understood locally, ignoring CCDs can therefore negatively impact or distort needs assessments, service uptake, quality of care, and effectiveness of programs [18, 29, 31]. In the South Asian context, Weaver [51] and Wahid and colleagues [50] have suggested screening for tension is a strategy to jumpstart conversations about mental health in a non-stigmatizing manner. How exactly CCDs are integrated into mental health assessments and interventions, however, differs considerably across studies. A recent review of the global mental health literature suggests that CCDs are most frequently integrated at the assessment, rather than the intervention level, with some researchers developing local assessments of CCDs while others integrating them into adaptations of pre-existing measures [10]. These locally developed and adapted measures have shown good reliability and validity. Examination of the symptoms associated with the five CCDs our study identified with commonly used mental health measures in this context–namely, the Harvard Trauma Questionnaire (HTQ, [37, 42], Hopkins Symptom Checklist (HSCL, [41], Patient Health Questionnaire-9 [30, 37], Generalized Anxiety Disorder-7 (GAD-7)–suggest that most, if not all, items present on the HSCL, PHQ-9, and GAD-7 are reflected in symptoms associated with the conditions. However, there are also symptoms present that are not captured by any of those measures, notably related to changes in social interactions (e.g., absence of happiness when with children, increased tendency to quarrel with family members) and various bodily pain (e.g., sharp body pain, neck pain). Where conceptually similar items were present, the language used, including metaphors evoked to express the experience of that symptom, may differ. These findings suggest that standardized measures as-is may omit or not prioritize symptoms that are more central to the lived experience of distress among this community. Future studies should either develop local screening measures for CCDs identified in this study or map and integrate their associated symptoms into an existing measure. Furthermore, future studies should examine the degree to which people may turn to MHPSS services for these CCDs. For instance, while one treatment or coping method for the other CCDs includes seeking input from or discussions with others, *feshar* is, instead, one participants thought should be individually managed.

This study has several key limitations that constrain the generalizability of findings. First, we had utilized a convenience sample, so findings may not reflect the experiences of other Rohingya refugees in the refugee camps. Second, utilization of Rohingya research staff may carry both challenges and benefits. Having staff that spoke the language and were themselves refugees living in the camps may have increased relatability of study staff and the willingness of participants to share more information. At the same time, the close cultural distance with the community may increase the likelihood participants either omit details that were considered shared or common knowledge or avoid topics that might be considered more socially stigmatizing. At the same time, because participants were speaking to wider beliefs within the community, rather than their personal experiences, this may limit the impact of social desirability on participants’ responses.

## Conclusion

Mental health burden continues to be high among Rohingya refugees in Bangladesh. Locally validated assessments are needed to accurately screen for mental health and psychosocial distress to identify need for intervention, and to monitor the effect of treatment. This study contributes to these efforts by investigating cultural concepts of distress among a community sample of Rohingya refugees. The concepts identified in this study do not neatly correspond onto standard measures of depression, anxiety, or Post-Traumatic Stress Disorder; yet are important and culturally salient ways in which people understand, experience, and communicate about the distress they experience in this context. Findings thus lay the groundwork for future efforts to advance locally developed or adapted measures and integrate CCDs into wider mental health and psychosocial support programming.

## Data Availability

The datasets used and/or analysed during the current study are available from the corresponding author on reasonable request.
